# Assessment of patients' and caregiver’s perceived need to start actively participated outcome measures in neurological research

**DOI:** 10.1186/1745-6215-16-S1-O2

**Published:** 2015-05-29

**Authors:** MG Celani, R Papetti, C Piersanti, S Macone, A Sgoifo, M Bianchi, A Bignamini, T Cantisani, K Mahan

**Affiliations:** 1 Azienda Ospedaliera Universitaria di Perugia, Perugia, Italy; 2 Cochrane Neurological Field - Health Authority of Umbria, Perugia, Italy; 3 Department of Pharmaceutical Sciences (DISFARM) - University of Milan, Milan, Italy

## Background

The Cochrane Neurological Field endeavours to create a research environment that reflects the needs of patients and caregivers, alongside health professionals, decision-makers, and including pharma. Collecting “the end-user of treatments” perspectives, ideas and values to reach agreement between these different partners will facilitate awareness of all stakeholder needs.

## Methods

The study was conducted in Umbria (Italy) on three adult populations affected by disabling neurological diseases: Epilepsy, Stroke and Dementia. It was structured through the use of Focus Groups. Patients and carers were recruited by telephone calls from the patients lists maintained by hospital wards, outpatients clinics and charities. From the respondents, two groups of patients and two groups of carers were formed for each level of severity of the disease based on the modified Rankin Scale (0-2; 3; 4-5).

Two psychologists conducted recruitment, used pre-determined semi-structured questions to interview attendees, and moderated group discussions. These were digitally recorded and transcribed into text-files, blindly analyzed and elaborated into key semantic meanings expressing perceived needs and emotions. Codes were analyzed using «Concordance» software, to identify the key semantic significants expressing the perceived needs and emotions in relation to the disease and its consequences.

## Results

Preliminary results of focus groups in Epilepsy are presented: 61 patients and carers participated (40% of people contacted), organized into 5 groups of patients, and 6 groups of Caregivers. Most frequently expressed needs were “assistance”, expressed 3 times more frequently by carers than patients, “Experience sharing” and “need for knowledge” expressed 2 times more frequently by patients. The need for assistance was directly proportional with disease severity, while the need for knowledge inversely proportional with disease severity.

Emotions most frequently expressed were anger and fear, proportional with disease severity, but also hope, resignation and acceptance. These two were 2 and 5 times each more frequently expressed by patients. Anger and hope were equally distributed among patients and carers.

## Conclusions

These preliminary data support the idea of a gap in research between different interest groups. Patient and carers’ priorities are based on intense personal insight, representing a starting point to work for shared outcome measures in clinical trials and shared agenda in research [1]. This could have a vast impact in general but may also reduce the burden of disease and represent significant savings in health care.
